# Cost-effectiveness of laser Doppler imaging in burn care in the Netherlands

**DOI:** 10.1186/1471-2482-13-2

**Published:** 2013-02-01

**Authors:** M Jenda Hop, Jakob Hiddingh, Carlijn Stekelenburg, Hester C Kuipers, Esther Middelkoop, Marianne K Nieuwenhuis, Suzanne Polinder, Margriet E van Baar

**Affiliations:** 1 Association of Dutch Burn Centres, Red Cross Hospital, Beverwijk, the Netherlands; 2 Burn Centre, Maasstad Hospital, Rotterdam, the Netherlands; 3 Burn Centre, Martini Hospital, Groningen, the Netherlands; 4 Burn Centre, Red Cross Hospital, Beverwijk, the Netherlands; 5 Department of Plastic, Reconstructive and Hand Surgery, MOVE Research Institute, VU University Medical Centre, Amsterdam, the Netherlands; 6 Department of Public Health, Erasmus Medical Centre, Rotterdam, the Netherlands

**Keywords:** Laser doppler imaging, Burns, Diagnosis, Cost-effectiveness analysis

## Abstract

**Background:**

Early accurate assessment of burn depth is important to determine the optimal treatment of burns. The method most used to determine burn depth is clinical assessment, which is the least expensive, but not the most accurate.

Laser Doppler imaging (LDI) is a technique with which a more accurate (>95%) estimate of burn depth can be made by measuring the dermal perfusion. The actual effect on therapeutic decisions, clinical outcomes and the costs of the introduction of this device, however, are unknown. Before we decide to implement LDI in Dutch burn care, a study on the effectiveness and cost-effectiveness of LDI is necessary.

**Methods/design:**

A multicenter randomised controlled trial will be conducted in the Dutch burn centres: Beverwijk, Groningen and Rotterdam. All patients treated as outpatient or admitted to a burn centre within 5 days post burn, with burns of indeterminate depth (burns not obviously superficial or full thickness) and a total body surface area burned of ≤ 20% are eligible. A total of 200 patients will be included. Burn depth will be diagnosed by both clinical assessment and laser Doppler imaging between 2–5 days post burn in all patients. Subsequently, patients are randomly divided in two groups: ‘new diagnostic strategy’ versus ‘current diagnostic strategy’. The results of the LDI-scan will only be provided to the treating clinician in the ‘new diagnostic strategy’ group. The main endpoint is the effect of LDI on wound healing time.

In addition we measure: a) the effect of LDI on other patient outcomes (quality of life, scar quality), b) the effect of LDI on diagnostic and therapeutic decisions, and c) the effect of LDI on total (medical and non-medical) costs and cost-effectiveness.

**Discussion:**

This trial will contribute to our current knowledge on the use of LDI in burn care and will provide evidence on its cost-effectiveness.

**Trial registration:**

NCT01489540

## Background

In patients with burns an early accurate diagnosis of burn depth is essential to determine the most appropriate treatment. Monstrey et al. recently reviewed all current modalities to diagnose burn depth. Bedside clinical examination is the most widely used and least expensive method for burn depth assessment. This technique is effective when diagnosing burns at the extreme end of the spectrum: superficial or full thickness. In partial thickness burns, however, clinical examination is not very accurate. Clinical burn depth assessment is accurate in about 2/3 of the cases, the most reported error is overestimation of depth [1].

Overestimation of burn depth can lead to unnecessary excision and grafting [2,3]. On the other hand, underestimation of burn depth may lead to an unnecessary delay in surgery, with a longer length of hospital stay and higher hospital costs as a consequence [4,5]. In burn care, traditionally classified as expensive care, there is a growing interest in costs and cost control [6,7]. In order to provide effective and cost-effective burn care, there is a need for an accurate method for burn depth estimation.

Laser Doppler imaging (LDI) is the only technique that has been shown to accurately predict wound depth with a large weight of evidence [1]. Laser Doppler imaging is based on the Doppler principle. Laser light that is directed at moving blood cells in sampled tissue exhibits a frequency change that is proportional to the amount of perfusion in the tissue. Laser Doppler imaging combines the advantages of laser Doppler and scanning techniques: the whole burn can be sampled and no direct contact with the burn surface is necessary [2]. In daily practice, LDI will be used in combination with standard clinical assessment [8], as a so-called add-on test [9].

Several prospective studies on the diagnostic accuracy of laser Doppler imaging have demonstrated an accuracy varying between 95-100% [3,8,10-12]. Timing of LDI is important: only scanning between 48 hours and 5 days results in a high accuracy (>95%) [8,11,13,14].

To decide whether a new diagnostic strategy, like LDI, should be implemented, assessment of diagnostic accuracy should be followed by assessment of diagnostic and therapeutic impact, effectiveness and cost-effectiveness of the new technology [9,15].

The literature on the accuracy of LDI in burn depth assessment is convincing. However, most studies only report on the accuracy of this technique. The diagnostic and therapeutic impact of the introduction of LDI is often only speculated upon.

There is, to our knowledge, only one retrospective cohort study [4], and one prospective non-randomized study [5] that investigated the therapeutic impact of the introduction of laser Doppler imaging. Petrie et al. reported a lower rate of operative interventions (6.8% before and 2.2% after, p= 0.029) in a pediatric burn population after the introduction of LDI and a reduced length of hospital stay of the surgical treated patients (15.1 days before and 9.8 days after, no p-value). The overall length of stay was 3.4 days in 235 patients before and 2.1 days in 270 patients after the introduction of the LDI [4]. Because of the retrospective nature of this study, other factors than the introduction of LDI alone could be responsible for the therapeutic changes [9]. In the study of Kim et al. [5] the impact of LDI on surgically treated pediatric patients with burns was investigated. The mean time to decision making for grafting procedures was shorter in the LDI group compared to the clinically assessed group (8.9 vs. 11.6 days, p= 0.01). Because of the non-randomised design, it is unclear whether this can be contributed to the LDI or to other differences between the groups.

Thus, current research gives some indications on the diagnostic and therapeutic impact of the LDI in burm care. However, randomised studies in both pediatric and adult patients with burns are lacking.

The introduction of LDI possibly leads to a cost reduction in burn care, by preventing unnecessary surgery [3,4], and reducing length of hospital stay [4]. However, no prospective studies are available on the costs and the possible cost reduction of LDI in burn care, nor are cost-effectiveness studies. Therefore, we can conclude that is it still unclear whether LDI actually influences diagnostic and therapeutic decisions, patient outcomes and costs, and thus adds to the quality of care.

The aim of our study is to analyse the effectiveness and cost-effectiveness of LDI in burn care. The effect of the use of LDI in burn care on decision making, on clinical outcomes, on costs, and on cost effectiveness will be assessed. The current diagnostic strategy in burn depth assessment (clinical assessment) is compared with the new diagnostic strategy: LDI in combination with clinical assessment. We expect that LDI in combination with clinical assessment can lead to earlier excision and grafting in Dutch burn care. With the results of this cost-effectiveness study, we aim to provide a guidance to decide whether this instrument should be implemented in Dutch burn care.

## Methods/design

### Study design

A multicenter, randomised controlled trial will be conducted in the three Dutch burn centres: Beverwijk, Groningen and Rotterdam.

### Participants

All consecutive patients of any ages with acute burns of indeterminate depth (assessed by the treating clinician), who are seen within 5 days post burn at one of the three burn centres, are eligible.

Inclusion criteria:

• Patients with acute burns of indeterminate depth (=intermediate depth; the burn wound is not obviously superficial or obviously full thickness)

• Outpatient treatment or admission in one of the three Dutch burn centres

• Presentation within 5 days post burn

Exclusion criteria:

• The presence of full thickness wounds, next to intermediate wounds

• Topical treatment/dressings that impair scanning (e.g. hydrocolloid dressings)

• Patients with peri-orbital facial burns, in which the eyes are unable to shield

• Patients or their next of kin if they are under aged or temporary incompetent who can not be expected to give informed consent e.g. because of cognitive dysfunction or poor Dutch proficiency.

• Patients with a TBSA burned > 20%

### Intervention

We will include a total of 200 patients and randomly divide them in two groups: new diagnostic strategy versus current diagnostic strategy. In the first few days the burns will be treated with regular topical antimicrobials or dressings. LDI is performed between 48 hours and 5 days post burn in this study. After removal of the topical agent (during regular wound care), all wounds of indeterminate depth are scanned by a trained research physician or nurse, who is not involved in the patient treatment. In case of clearly superficial wounds next to intermediate wounds in a study patient, the superficial wounds will not be scanned; similar to what would happen in daily practice.

**Figure 1:**
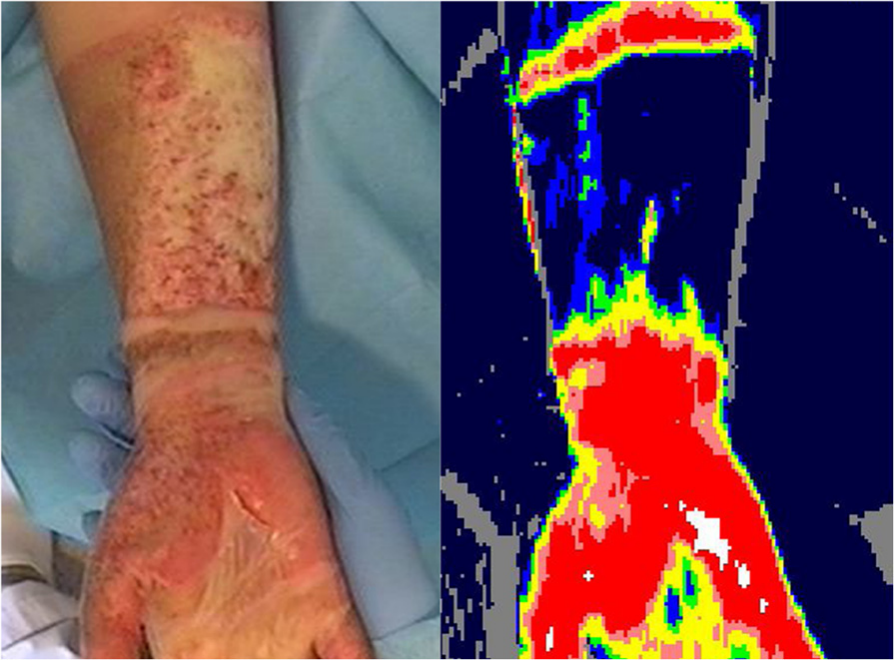
**Clinical appearance of a three-days-old flame burn in a woman of 21.** The LDI scan shows a burn with a healing potential < 14 days of the right hand palm and a healing potential > 21 days of the right lower arm. Written consent was obtained from the participant to publish this figure.

Results of LDI are (Figure 1): [12]

• Red/pink represents a healing potential within 14 days post burn

• Yellow/green represents a healing potential between 14–21 days

• Blue represents a healing potential > 21 days

In the group ‘new diagnostic strategy’, the results of the LDI scan will be revealed to the treating clinician. Subsequent treatment and outcome of this group will be compared to that of the group with ‘current diagnostic strategy’ in which the treating clinician is blinded for the results of the LDI scan.

### Outcomes

#### Primary outcome measure: wound healing time

Wound healing time is defined as the number of days between LDI (randomisation) and the day on which reepithelialisation of >95% is achieved. Next to that, for generalisation and LDI accuracy checks, we will present the time to wound healing from the day of injury. During wound care an experienced burn specialist will assess reepithelialisation; this is a reliable and valid technique (compared to digital image analysis) [16]. Wounds in admitted patients are assessed daily and wounds in outpatients two or three times a week. Differences in wound healing time between the two randomisation groups will be analysed.

#### Secondary outcomes measures

##### Quality of life and scar quality

Quality of life will be measured as soon as possible after injury to determine pre-injury functioning (at least within one month post burn) and after 3 months with the EuroQol-6D (EuroQol-5D + cognitive functioning; validated in patients aged ≥ 5 years) or the ItQol-47 questionnaire (validated in children < 5 years of age) [17-19].

The EuroQol-5D questionnaire can result in 243 different health states, which can be converted in a summary (utility) score between 0 (death) and 1 (perfect health) [18]. The EuroQol (EQ-5D) is an extensively used general health questionnaire to measure quality of life. It is recommended for the assessment of HRQoL in injury patients, especially for economic assessments [20].

Scar quality will be measured 3 months post-injury. Scar quality will be measured as follows;

• Elasticity will be measured with the Cutometer® Skin Elasticity Meter 575 (Courage and Khazaka Electronic GmbH, Cologne, Germany) The cutometer provides several elasticity parameters. In this study, maximal skin extension (Uf) (in mm) will be used, because this has been demonstrated to be the most reliable parameter [21].

• Vascularity and pigmentation will be measured with the Dermaspectometer, which is a reliable narrowband spectrometer that computes an erythema and melanin index (Cortex Technology, Hadshund, Denmark) [22].

• Subjective scar assessment will be performed by means of the Patients Observer Scar Assessment Scale (POSAS), a reliable and valid scar assessment tool that consists of a patient and observer scale [22,23]. The patient scores the scar characteristics colour, pliability, thickness, relief, itching and pain. The observer scale contains the items vascularisation, pliability, pigmentation, thickness, and relief. All items are scored numerically on a 10-point rating scale. In addition, the two observers and the patient give a general opinion on the scare quality on a 10-point rating scale.

Differences in quality of life and scar quality 3 months post burn between the two diagnostic groups will be analysed.

##### Diagnostic decisions: diagnostic effect of LDI and accuracy

In the ‘new diagnostic strategy’ group the diagnostic effect of the introduction of LDI will be assessed on the scanning day by comparing diagnostic decisions of burn clinicians, just before and after the use of LDI.

Possible diagnostic decisions before LDI are:

• Superficial partial thickness burn (no study wound)

• Intermediate burn, the burn wound is not obviously superficial or full thickness (if parts of a burn wound are obviously full thickness, this will be recorded also)

• Deep partial thickness of full thickness burn (exclusion patient from study)

Possible diagnostic decisions after LDI are [12]:

• Superficial partial thickness burn, will heal within 14 days

• Intermediate partial thickness burn, will heal between 14–21 days (the spectrum of an ‘intermediate burn’ is smaller after the use of LDI compared to before the use of LDI)

• Deep partial thickness or full thickness burn, will not heal within 21 days

Accuracy of LDI in our study patients will be checked also. Results of laser Doppler imaging are compared with the reference tests: time of wound healing (from burn date until >95% reepithelialisation) and biopsies. In our study protocol, wounds with a healing potential ≤ 21 (assessed by the LDI) are considered as superficial or intermediate partial thickness and wounds with a healing potential > 21 days are considered as deep partial thickness or full thickness. Only in case of surgery a 3 mm-punch biopsy will be taken as a reference test to assess burn depth [1]: in a superficial burn the basal membrane is partially destroyed; in a deep partial thickness burn the basal membrane is entirely destroyed and the dermis partially destroyed; in a full thickness burn, the dermis is also completely destroyed.

##### Therapeutic decisions: timing of surgery indication, timing of surgery and length of hospital stay

The effect of LDI on therapeutic decisions by the burn clinician will be assessed. The first therapeutic decision is on admittance, the second monitored decision is after 2–5 days (based on clinical assessment or clinical assessment in combination with LDI result). In case of postponement of decision, reason of postponement will be investigated and decision making is followed until wound healing.

The possible therapeutic decisions are:

• Surgery

• Postponement of decision

• No surgery

Differences in therapeutic decisions on the day of randomisation will be analysed.

In case of surgery, differences in timing of surgical decision and timing of surgery (in days after randomisation) between the two diagnostic groups will be analysed.

Differences in length of hospital stay between the two diagnostic groups will be analysed as well.

##### Economic evaluation: total costs and cost-effectiveness

The economic evaluation will be performed from a societal perspective in accordance with the Dutch guidelines for economic evaluation studies [24]. A cost-effectiveness analysis will assess the balance between costs and effects of the new diagnostic strategy compared to the current diagnostic strategy.

Total costs in this study represent direct healthcare costs (inpatient and outpatient medical costs), direct non-healthcare costs (travel costs) and indirect non-healthcare costs (productivity loss). Real medical costs will be calculated by multiplying the volumes of health care use with the corresponding unit prices. Costs will be calculated from randomisation until 3 months post burn. A bottom up approach will be applied (following the micro-costing method of Gold et al. 1996) based on a detailed inventory of all resource used during admittance in one of the Dutch burn centres [25].

Cost prizes will be inventoried in the financial department of one burn centre and will be used for all centres in order to prevent measuring cost differences between burn centres. The costs apply to the financial year 2012.

*Specialized burn care costs* include intervention costs, hospital days, and other variable costs:

• The costs per unit of the LDI scanning will be determined by taking into account the initial investment of equipment, investments during use, maintenance, numbers of years of use, discounting, number of procedures per year and personnel costs.

• Hospital day (both ICU and non ICU) costs will be calculated by multiplying the length of hospital stay by the costs of one day of admission. The cost of re-admittance during follow-up will also be calculated. The costs of a day of admittance are based on a detailed inventory (following the micro-costing method of Gold et al. 1996) of fixed costs only: staffing costs, accommodation, equipment, overhead, food, laundry, and medication [25].

• Variable hospital costs will be identified per patient and calculated by multiplying the volumes of health care use with the corresponding unit prices. The included items are: dressing costs, surgical procedures (material, equipment and personnel costs), diagnostic procedures (bronchoscopy, swabs, laboratory and radiology costs), treatment by allied health professionals, splints, pressure garments, and outpatient visits (staffing and material costs).

Data registration will start on the day of randomisation. Data regarding patients’ baseline characteristics and health-care use will be obtained from patient records and the electronic hospital administration.

*Other healthcare costs and non healthcare costs* will be calculated based on charges as a proxy of real costs [24] with the help of data obtained from patient questionnaires after 3 months. Other healthcare costs include nursing-home and rehabilitation centre, homecare, visits to general physicians and allied health professional outside the hospital. Indirect non-healthcare costs include productivity loss in patients and partners or parents (if applicable), and direct non-healthcare costs include patient travel costs.

To measure the economic impact of LDI in burn care cost-effectiveness will be assessed by calculating the incremental cost-effectiveness ratio, defined here as the costs for the ‘new diagnostic strategy’ (minus savings) divided by the difference in wound healing time (measured from randomisation) between the ‘new diagnostic strategy’ and ‘current diagnostic strategy’. Secondary, a cost-utility analysis will be performed, i.e., as cost per Quality Adjusted Life Years (QALY). The cost-utility ratio can be calculated in patients ≥ 5 years only. In children under the age of 5 years, the EuroQol-5D is not validated and no questionnaires are available that allow the calculation of utility scores [18,19]. Overall utility scores for population-based quality of life (derived from the EuroQol-5D) will be obtained and expressed as QALY’s. QALY’s will be calculated by multiplying the utility of a health state by the time spent in this health state using the Dutch valuation tariff [26]. Policymakers and health economists have proposed that costs varying from €25,000 up to €75,000 per QALY may be considered as acceptable [27]. In accordance with guidelines for differential discounting, effects will be discounted at a rate of 1.5% and costs at 4% per year [24].

In case no differences will be found in wound healing and quality of life, the economic evaluation will be based on a cost-minimisation analysis, that consists of a comparison of total costs in both diagnostic strategies.

### Sample size

A total of 57.5% of admitted burn patients in the Netherlands is treated conservatively (unpublished data, Dutch Burn Repository R3, 2011). The time to wound healing in patients without surgery is approximately 13 days, this will not change after the introduction of LDI. In the 42.5% of surgically treated patients we do expect an effect on wound healing time, because of an earlier operation. The mean time to first transplantation is 14.0 days post burn, the mean time to wound healing is 5 days after surgery [28], resulting in a mean time to wound healing of 19.0 days. The overall mean time to wound healing for all patients combined is 15.6 days ((57.5%*13+ 42.5%*19)/100).

Petrie et al. describe a reduction of 5.3 days in LOS in patients undergoing surgery, after the introduction of the LDI. As a result, in our population, an overall effect of 2.25 (57.5%* 5.3) days can be expected, with a standard deviation of 5 days. A 2-sided test with an α level of 0.05 and a β level of 0.20 (power 0.80) indicates a required total sample size of 190 patients. We expect a low drop out rate because of a short time to follow up to assess primary outcome (time to wound healing). With a 5% drop out we need a total sample size of 200.

## Randomisation

Patients are randomly allocated to the group ‘new diagnostic strategy’ or the group ‘current diagnostic strategy’, on the day of the LDI scan. Stratification will be performed for the three different centers and the severity of the burns: total burned surface area ≤10% or >10%. A randomisation list is prepared for each stratum on the principle of random permuted blocks of patients, using a random number table [29]. The allocation sequence is concealed by using sequentially numbered, opaque sealed envelopes (SNOSE) prepared by the coordinating researcher (MJH) which are opened at day of LDI after having obtained informed consent, by one of the local researchers [30].

The care provider, outcome assessor and patients are blinded to the results of the LDI in the group ‘current diagnostic strategy’. However, they are not blinded to the group in which patients are randomised.

## Statistical analysis

Data will be primarily analysed according to the intention-to-treat principle. We assume that time to LDI will be equal in both diagnostic groups, as a result of randomisation. Thus, the differences in effects (e.g. time to wound healing) and costs will be assessed from randomisation onwards.

Differences in time to wound healing (numbers of days between LDI and day of > 95% reepithelialisation) and timing of surgery in both diagnostic strategy groups will be analysed with Kaplan-Meier curves and log rank test.

Quality of life (derived from the ItQol-47 and EuroQol-6D), scar quality (assessed by the Cutometer®, Dermaspectometer® and POSAS) and length of hospital stay will be analysed using the independent-sample-t-test (in case of normal distribution) or the Mann–Whitney test (non-normal distribution).

Differences in diagnostic and therapeutic decisions will be analysed with the Chi-square test. Diagnostic accuracy will be assessed by calculating sensitivity and specificity of the LDI compared to the reference test: time to wound healing (in conservative treated patients) or biopsy (in surgical treated patients). In addition, receiver operating characteristic (ROC) curves will be calculated for both diagnostic groups.

Differences in mean costs (after randomisation) will be analysed by the Mann–Whitney test, since healthcare costs are typically highly skewed. Non-parametric techniques (bootstrapping) will be used to derive a 95% confidence interval for the differences in distributions of the costs.

The cost-effectiveness analysis and cost-utility analysis are performed by dividing the differences in average costs by the differences in average effects or utility. In a sensitivity analysis, the impact on cost-effectiveness of statistical uncertainty on the main study outcomes will be determined (uni- and multivariable).

Data analysis will be performed using SPSS-software (Statistical Package for the Social Sciences).

## Discussion

In this paper we described the design of our study into the effects and cost-effectiveness of laser Doppler imaging in Dutch burn care. This is the first randomised controlled study that analyses not only the accuracy, but also the effects and costs of the introduction of LDI in burn care. Strengths of our study are the extensive cost calculations (not only LDI costs are included) and the detailed analysis of the possible impact of the introduction of the LDI in terms of process changes, i. e. changes in diagnosis and therapy. A limitation of this study is that the cost calculations will be performed in Dutch burn centres only. Although the multicentre analysis improves the generalisability of the cost distribution, compared to a monocentre analysis, total cost and cost distribution in other countries are probably different [31].

Another limitation of this study is that it tests the effectiveness of the introduction of LDI, so there’s a possible learning effect in the study. In the first patients, professionals will less likely rely on LDI and adapt their therapy. As a result, differences between randomisation groups will probably be smaller, and subsequently our study underestimates the full effect of the introduction of LDI in burn care.

Next to a time effect, there will probably also be an effect of differences in study sites. The usual care differs between study sites; this can influence the results of the introduction of LDI in the burn centres. These possible differences will be studied by providing sub-analyses per burn centre.

This study will undoubtedly contribute to our knowledge about the use of LDI in burn care and will provide evidence on its cost-effectiveness. Inclusion of patients started in December 2011 and presentation of data will be expected at the end of 2013.

## Ethical considerations

This study has been approved by the Medical Research Ethics Committee of Rotterdam (NL37844.101.11).

## Competing interests

The authors declare that they have no competing interests. The laser Doppler imagers were leased from Moor Instruments for this study.

## Authors’ contributions

MJH made substantial contributions to conception and design, drafted the manuscript, and contributes currently to acquisition of data. JH made substantial contributions to conception and design, revised the manuscript critically, and contributes currently to acquisition of data. CS revised the manuscript critically and contributes currently to acquisition of data. HCK revised the manuscript critically and contributes currently to acquisition of data. EM made substantial contributions to conception and design and revised the manuscript critically. MKN made substantial contributions to conception and design and revised the manuscript critically. SP made substantial contributions to conception and design and revised the manuscript critically. MEB made substantial contributions to conception and design and revised the manuscript critically. All authors read and approved the final manuscript.

## Pre-publication history

The pre-publication history for this paper can be accessed here:

http://www.biomedcentral.com/1471-2482/13/2/prepub
